# Supplementary material for: Unravelling the role of epigenetic regulators during embryonic development of Rhipicephalus microplus
Source: bioRxiv. 2025 Jul 11:2025.07.11.662657. Preprint. [Version 1] doi: 10.1101/2025.07.11.662657 (PMC12265629; doi:10.1101/2025.07.11.662657)
Supplement: Supplement 1 — Supplemental Figure 1. Protein sequence alignment of full-length epigenetic regulators from Rhipicephalus microplus (Rm), human (Hs), and bovine (Bt). Amino acids highlighted in grey represent conserved residues, while those in black indicate identical residues across species. [file media-1.pdf]

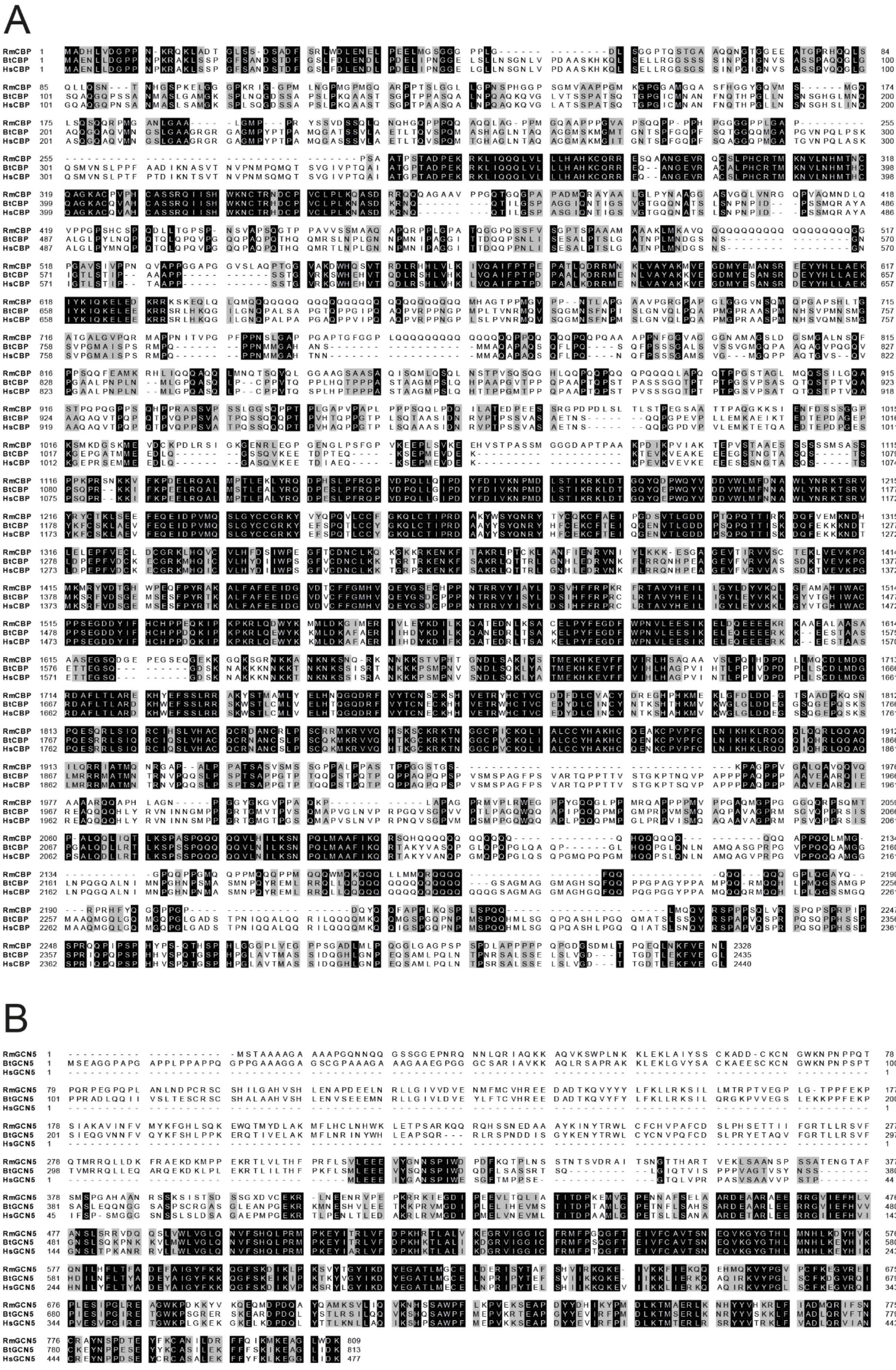

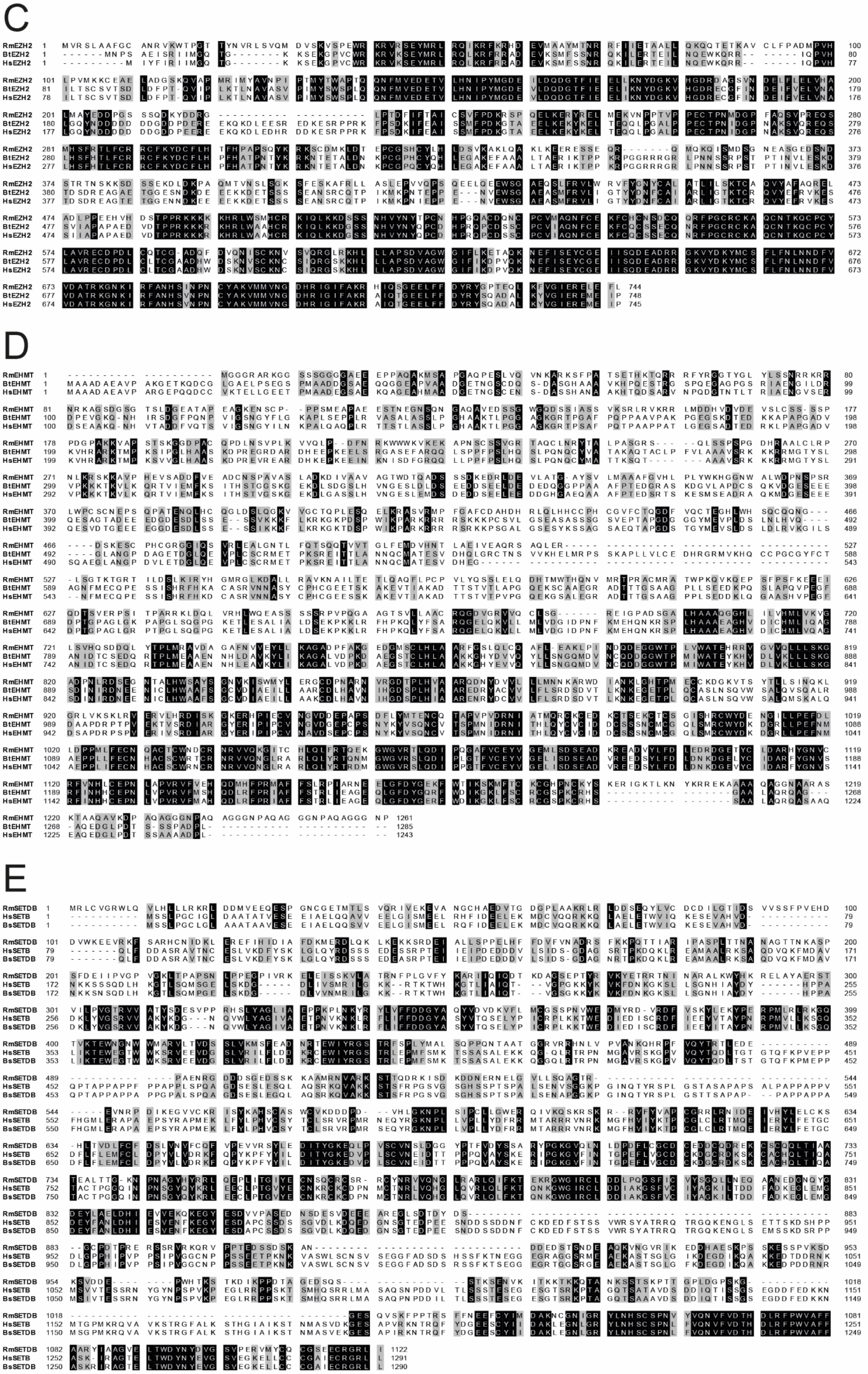
C
RmEZH2
BtEZH2
HsEZH2
D
RmEHMT
BtEHMT
HsEHMT
E
RmSETDB
HsSETB
BsSETDB

F

```
RmSETD2 1    ---------- -MKEKAECNN LEETKAQNGH SSHITNGSTP NESDSSVLDS SGTSS----- ---------- ---------D SPTSSVSPSS AQQDPERLHI 65
HsSETD2 1    MEFSIKQSPL SVQSVVKCIK MKQAPEILGS ANGKTPSCEV NRECSVFLSK AQLSSSLQEG VMQKFNGHDA LPFIPADKLK DLTSRVFNGE PGAHDAKLRF 100
BtSETD2 1    MEFSVRKSPL SAPKVVKCVK MKQAPEVLGS ANGKTPSCDV SRECSVFLSK AQLSAGLQDG VVQKFNGHDA LPFIPAERLK DLTSRVFNGE PGAHDAKLRF 100

RmSETD2 66   ARRSKFGRVR KPKVNPCMEY TP-----IVR GYS------- ---------- -SHNRAKQGT PSH------- ---------- ---------- --SPPSPSHG 113
HsSETD2 101  ESQEMKGIGT PPNTTPIKNG SPEIKLKITK TYMNGKPLFE SSICGDSAAD VSQSEENGQK PENKARRNRK RSIKYDSLLE QGLVEAALVS KISSPSDKKI 200
BtSETD2 101  ESQEVKGLGT PPHTTPVKNG SPEIKLKITK TYMNGKPLFE SSICGDGAAA VSHSEADGQR PEYKARRSRK RSAQRGPVLE HGLGEAALAS RLSSPVDKKI 200

RmSETD2 114  GETPTSVSTA ASEPTQSSQY LVGDLLWVKV GNHPHWPTMV SYDPVSGTHT KVVRNK---R IYHVQYFGTA VQHGWTQPNR TLPFEGLEKF MELIDSG--- 207
HsSETD2 201  PAKKESCPNT GRDKDHLLKY NVGDLVWSKV SGYPWWPCMV SADPLLHSYT KLKGQKKSAR QYHVQFFGDA PERAWIFEKS LVAFEGEGQF EKLCQESAKQ 300
BtSETD2 201  PVKRESCPNS SRDKGQLLKY DVGDLVWSKV SGYPWWPCMV SADPLLHSHT RLKGQKKSAR QYHVQFFGDA PERAWIFEKS LVAFEGEGQF EKLCQESAKQ 300

RmSETD2 207  --------RV PHGVPPRKRD DWKVAVEEAV AAMQMTRAQR KLQLTFKYY- ---------- ---------- ---------- ---------- ---------- 248
HsSETD2 301  APTKAEKIKL LKPISGKLRA QWEMGIVQAE EAASMSVEER KAKFTFLYVG DQLHLNPQVA KEAGIAAESL GEMAESSGVS EEAAENPKSV REECIPMKRR 400
BtSETD2 301  APTKAEKIKL LKPISGKLRA QWEMGLVQAE AAARMAVEER KAKFTFLYVG DQLRLNPHVA KEAGVAVESL GEAAEPSGVE EEAADSPKSS GEEGAPVKRR 400

RmSETD2 248  ---------- ---------- ---------- ------DPPS KK-------- ---------- ---------- ---------- ---------- ---------- 254
HsSETD2 401  RRAKLCSSAE TLESHPDIGK STPQKTAEAD PRRGVGSPPG RKKTTVSMPR SRKGDAASQF LVFCQKHRDE VVAEHPDASG EEIEELLRSQ WSLLSEKQRA 500
BtSETD2 401  RRAKLSSSTE NLPGDPGPGR ATPQKVAEAD SRRGAGSPPG RKKAAASTPR SRRGDAASQF LVFCQKHRDE VVAEHPDASA EEIEELLASQ WNMLNEKQKA 500

RmSETD2 254  ---------- ---------- ---------- ---------- ---------- ---------- ---------- ---------- LKSEHPPK-- ---------- 262
HsSETD2 501  RYNTKFALVA PVQAEEDSGN VNGKKRNHTK RIQDPTEDAE AEDTPRKRLR TDKHSLRKRD TITDKTARTS SYKAMEAASS LKSQAATKNL SDACKPLKKR 600
BtSETD2 501  RYHTKFALVA PPQSEEDSGN LNGKKRSHTK RTQGLRGDAE VEEAPRKRLR TDRHGLRKRE TITDKTARTT SCKALEAASS LKSQAATKHL SDACKPLKKR 600

RmSETD2 262  ---------- ---------- ----ENGVDQ EATEKN---- ---------- ---------- ---------C LICGASGATL TCTGTCGMSF HLGCIGITQP 305
HsSETD2 601  NRASTAASSA LGFSKSSSPS ASLTENEVSD SPGDEPSESP YESADETQTE VSVSSKKSER GVTAKKEYVC QLCEKPGSLL LCEGPCCGAF HLACLGLSRR 700
BtSETD2 601  HRAPAAAATT LTFSKSSSPS ASLTENEVSD GPGDEPPESP DESADETQTE ASVSSKRSER GATARKEHVC QLCEEPGSLV LCEGPCCGAF HLACLGLSRR 700

RmSETD2 306  PD-AFLCDEC TAGSHPCLVC NRTEATQK-C SMEQCGTFYH MSCLKTLPLP VKED-SPMCP AHFCLPCVQQ KP-RAVSMRG RLLRCVQCPS AFHP--ECLP 399
HsSETD2 701  PEGRFTCSEC ASGIHSCFVC KESKTDVKRC VVTQCGKFYH EACVKKYPLT VFESRGFRCP LHSCVSCHAS NPSNPRPSKG KMMRCVRCPV AYHSGDACLA 800
BtSETD2 701  PEGRLLCGEC TSGIHSCFVC KESKSDVKRC VVSQCGKFYH EACVRRFPLT VFESRGFRCP LHSCLSCHAS NPSNPRPSKG KMLRCVRCPV AYHGGDACLA 800

RmSETD2 400  AGSNTLGSSS LVCPRHGSQN ----TASAIN VSWCMVCSEG GKLLCCEGCP AAFHETCLGL QETPEGTFLC PDCQDWKYPK YGDMIWVKLG CYRWWPAQVC 495
HsSETD2 801  AGCSVIASNS IICTAHFTAR KGKRHHAHVN VSWCFVCSKG GSLLCCESCP AAFHPDCLN- IEMPDGSWFC NDCRAGKKLH FQDIIWVKLG NYRWWPAEVC 899
BtSETD2 801  AGCSVIASNS IICTNHFTAR KGKRHHAHVN VSWCFVCSKG GSLLCCESCP AAFHPDCLS- IEMPDGSWFC NDCRAGKKLH FQDIIWVKLG NYRWWPAEVC 899

RmSETD2 496  APGDIPLNIQ ALKHEPIGDF AVRFYGSHDY YWTNRKRVFL FEEGDSGSLN ASSKSVARLF QAALEEARQA WEE---QRRA RELGE---KP APFRMIRVNR 589
HsSETD2 900  HPKNVPPNIQ KMKHE-IGEF PVFFFGSKDY YWTHQARVFP YMEGDRGSRY QGVRGIGRVF KNALQEAEAR FREIKLQREA RETQESERKP PPYKHIKVNK 998
BtSETD2 900  HPKNVPPNIQ KMKHE-VGEF PVFFFGSKDY YWTHQARVFP YMEGDRGSRY QGVRGIGRVF KNALQEAEAR FREIKLQREA RETQESERRP PPYKHIKVNK 998

RmSETD2 590  VVCPGAAMPS AAVHKRSVCT CTPQD--PCR AN--CLNRLL LYECSRKLCP AGPACENQRF QKRTYAETAL VRTPGRGWGL LTSQALAAGD FVMEYVGELI 685
HsSETD2 999  PYG-KVQIYT ADISEIPKCN CKPTDENPCG FDSECLNRML MFECHPQVCP AGEFCQNQCF TKRQYPETKI IKTDGKGWGL VAKRDIRKGE FVNEYVGELI 1097
BtSETD2 999  PYG-KVQVHT ADISEIPKCN CKPTDENPCG SDSQCLNRML MFECHPQVCP AGESCQNQCF TKRQYPETKI VRTDGKGWGL VAKRDIRKGE FVNEYVGELI 1097

RmSETD2 686  DDKECERRLK ELHTDNIQNF YFLTLAKDRI IDAGPMGNMS RFINHSCQPN CVTQKWTVNG DTRVGIFAIR DIPEGTELTF NYNLECRGNE RIACACGSEN 785
HsSETD2 1098 DEEECMARIK HAHENDITHF YMLTIDKDRI IDAGPKGNYS RFMNHSCQPN CETLKWTVNG DTRVGLFAVC DIPAGTELTF NYNLDCLGNE KTVCRCGASN 1197
BtSETD2 1098 DEEECMARIK RAHENDITHF YMLTIDKDRI IDAGPKGNYS RFMNHSCQPN CETLKWTVNG DTRVGLFAVC DIPAGTELTF NYNLDCLGNE KTVCRCGASN 1197

RmSETD2 786  CSGYIGLRPR P----PAECE GKPSHSKSRP KKASD----- ---------- ---------- ---------- ---------- ---------- ---------- 816
HsSETD2 1198 CSGFLGDRPK TSTTLSSEEK GKKTKKKTRR RRAKGEGKRQ SEDECFRCGD GGQLVLCDRK FCTKAYHLSC LGLGKRPFGK WECPWHHCDV CGKPSTSFCH 1297
BtSETD2 1198 CSGFLGDRPK TSAPLSSEEK GKKTKKKARR RRTKGEGKKP SEDECFRCGD GGQLVLCDRK SCTKAYHLPC LGLGKRPFGK WECPWHHCDV CGKPSTAFCH 1297

RmSETD2 816  ---------- ---------- ---------- ---------- ---------- ---------- -------- 816
HsSETD2 1298 LCPNSFCKEH QDGTAFSCTP DGRSYCCEHD LGAASVRSTK TEKPPPEPGK PKGKRRRRRG WRRVTEGK 1365
BtSETD2 1298 FCPNSFCKDH QDTAAFSSTQ DGRLYCSEHD FRAEPARSAQ TEQPCPGPSK AKGRRRRRRC WRRVMEGE 1365
```

G

```
RmSETD4 1    -------MAK KGRNHRKKAR RRADQVSCTE DVSVDLLKWM TAKGFQLHTQ LYVKEFRETG RGLATQQMLS AGDPFLRLPT CLLITTGTAL SSSLHDFVIR 93
BtSETD4 1    MKNGGGRTSR IRRRKLFTSS ESRGVNESYK PEFIELKKWL KDRRFE-DTT LIPAHFPGTG RGLMSKTSLQ EGQTIISLPE SCLLTTDTVI RSYLGAYIAK 99
HsSETD4 1    ---------- ---------- ----MNESHK SEFIELRKWL KARKFQ-DSN LAPACFPGTG RGLMSQTSLQ EGQMIISLPE SCLLTTDTVI RSYLGAYITK 75

RmSETD4 94   NHRKLTPIEV LTLFLMNEKL RGHDSEWRYF INSLPTTYTT PVYLGSKLLA KLPCSVFRKA QTQVSKIRGT FLKLRALLKE NEDVDSLFAS LSKNFTWCLF 193
BtSETD4 100  WQPPPSPLLA LCTFLVSEKH AGDRSPWKPY LEVLPKAYTC PVCLEPEVVN LLPNPLKTKA WEQRSHVWEF FSSSRGFFSS LQ--PLFSEA VETIFSYRAL 197
HsSETD4 76   WKPPPSPLLA LCTFLVSEKH AGHRSLWKPY LEILPKAYTC PVCLEPEVVN LLPKSLKAKA EEQRAHVQEF FASSRDFFSS LQ--PLFAEA VDSIFSYSAL 173

RmSETD4 194  VWAWSAVNTR CIFSEHKTGN FLWDN-DKCA LAPFLDCLNH HWKASVQTAI VGS--YFEIV TNNNYQPNEQ VFISYGSHDN RKLLLEYGFV LPNNPNDLVI 290
BtSETD4 198  RWAWCAVNTR AVYMKRPPLL CLSPEPDTCA LAPYLDLLNH SPDVQVKAAF NEETRCYEIR TATRCGKHKE VFICYGPHDN HRLLLEYGFV CVSNPHACVY 297
HsSETD4 174  LWAWCTVNTR AVYLRPRQRE CLSAEPDTCA LAPYLDLLNH SPHVQVKAAF NEETHSYEIR TTSRWRKHEE VFICYGPHDN QRLFLEYGFV SVHNPHACVY 273

RmSETD4 291  ITREHLLKLH SWTTLPIPNF SSKLSFLEER NILSESCGFT MEGMTWNGKI AVKVLSHPEA SRSAWNSLLF LYGEDELDEQ E--------C QLVLTLVEAM 382
BtSETD4 298  VSKDILVKYL PSTAKQMN-- -KKISILEDH DFTEN-LTFG WDGPSWRLLT ALKLLCLEAE EFTCWKKVLL GEIISDTNEK ASLDVAQKIC HYFIEETHAV 393
HsSETD4 274  VSREILVKYL PSTDKQMD-- -KKISILKDH GYIEN-LTFG WDGPSWRLLT ALKLLCLEAE KFTCWKKVLL GEVISDTNEK TSLDIAQKIC YYFIEETNAV 369

RmSETD4 383  LCDYDAPLPE GKDLCSQT-- VATFIKEETS ILTKQIERYT I------ 421
BtSETD4 394  LRKVSRMKDE EVALMNQLTL VETLRTEELK ILQASAKALT TLQTAFS 440
HsSETD4 370  LQKVSHMKDE KEALINQLTL VESLWTEELK ILRASAETLH SLQTAFT 416
```

H

```
RmSETD7 1    MVKGRKKCAT NPTRSKKSAP ASCRSGDVNA SVKQQDGKST VASATRTSPP VTRSTRIDDY FPVLGAEMAT AEEVASEHSY SRRRPIKREP PCPLQQLVEP 100
BtSETD7 1    ---------- ---------- ---------- -------MSP NKCSGMRSPL QEENSVAQYE VKCQGKPLAG ---------I YRKRDEKRNS GNAIRSSMKA 54
HsSETD7 1    MARGRK---- ------MSKP RTDGENVFTG QSKIYSYMSP NKCSGMRFPL QEENSVTHHE VKCQGKPLAG ---------I YRKREEKRNA GNAVRSAMKS 81

RmSETD7 101  EVQIPCPDAA ALPAGLSLTA TQPPSPPSVD TKTPAVVVHR TYQDRSKSAS RATNKPATKV TVKAPVAAEH DPLKVAKRKT EVPCHSLTEY FSIRRSGRKT 200
BtSETD7 55   EEQK------ ------IKDA RRGPLAPFPN QKSEAAEPPK TPT----SSC DTPNAAAAKQ GLKKPVRGKQ APRKKAQGKT QQ-NRKLTDF YPVRRSSRKS 137
HsSETD7 82   EEQK------ ------IKDA RKGPLVPFPN QKSEAAEPPK TPP----SSC DSTNAAIAKQ ALKKPIKGKQ APRKKAQGKT QQ-NRKLTDF YPVRRSSRKS 164

RmSETD7 201  KATLVKEKQK QVEDAILNGI EEGFQVVELA DKGRGVTTSR PLKAGDFVLE YAGELIDVQE AKLREQIYAT DSSIGCYMYY FTCRNKQYCV DATKETGRLG 300
BtSETD7 138  KAELQSEERK RIDELIESGK EEGMKIDLID GKGRGVIATK QFSRGEFVVE YHGDLIEITD AKKREALYAQ DPSTGCYMYY FQYLSKTYCV DATRETNRLG 237
HsSETD7 165  KAELQSEERK RIDELIESGK EEGMKIDLID GKGRGVIATK QFSRGDFVVE YHGDLIEITD AKKREALYAQ DPSTGCYMYY FQYLSKTYCV DATRETNRLG 264

RmSETD7 301  RLVNHSKRGN LKTQTCIIKG VPHLVLVAQR NIEAGEELLY DYGDRSKASL QFHPWLAL 358
BtSETD7 238  RLINHSKCGN CQTKLHDIDG VPHLILIASR DIEAGEELLY DYGDRSRASI EAYPWLKH 295
HsSETD7 265  RLINHSKCGN CQTKLHDIDG VPHLILIASR DIAAGEELLY DYGDRSKASI EAHPWLKH 322
```

I

```
RmHDAC1 1    MAATMHSKKR VCYYYDGDIG NYYYGQGHPM KPHRMRMTHN LILNYGLYRK MEIYRPHKAT QEEMTKYHSD DYIRFLRSIR PDNMSEYNKQ MQRFNVGEDC 100
BtHDAC1 1    MAQTQGTKRK VCYYYDGDVG NYYYGQGHPM KPHRIRMTHN LLLNYGLYRK MEIYRPHKAN AEEMTKYHSD DYIKFLRSIR PDNMSEYSKQ MQRFNVGEDC 100
HsHDAC1 1    MAQTQGTRRK VCYYYDGDVG NYYYGQGHPM KPHRIRMTHN LLLNYGLYRK MEIYRPHKAN AEEMTKYHSD DYIKFLRSIR PDNMSEYSKQ MQRFNVGEDC 100

RmHDAC1 101  PVFDGLYEFC QLSTGGSVAG AVKLNKQATD IAVNWAGGLH HAKKSEASGF CYVNDIVLAI LELLKYHQRV LYIDVDIHHG DGVEEAFYTT DRVMTCSFHK 200
BtHDAC1 101  PVFDGLFEFC QLSTGGSVAS AVKLNKQQTD IAVNWAGGLH HAKKSEASGF CYVNDIVLAI LELLKYHQRV LYIDIDIHHG DGVEEAFYTT DRVMTVSFHK 200
HsHDAC1 101  PVFDGLFEFC QLSTGGSVAS AVKLNKQQTD IAVNWAGGLH HAKKSEASGF CYVNDIVLAI LELLKYHQRV LYIDIDIHHG DGVEEAFYTT DRVMTVSFHK 200

RmHDAC1 201  YGEYFPGTGD LRDIGAGKGK YYAVNFPLRD GIDDESYESI FQPLISKVME MYQPSAVVLQ CGADSLSGDR LGCFNLTLKG HGKCAEFVRR YNLPLLQLGG 300
BtHDAC1 201  YGEYFPGTGD LRDIGAGKGK YYAVNYPLRD GIDDESYEAI FKPVMSKVME MFQPSAVVLQ CGSDSLSGDR LGCFNLTIKG HAKCVEFVKS FNLPMLMLGG 300
HsHDAC1 201  YGEYFPGTGD LRDIGAGKGK YYAVNYPLRD GIDDESYEAI FKPVMSKVME MFQPSAVVLQ CGSDSLSGDR LGCFNLTIKG HAKCVEFVKS FNLPMLMLGG 300

RmHDAC1 301  GGYTIRNVAR CWTYETAVAL GVDIANELPY NDYFEYFGPD FKLHISPSNM ANQNTPEYLE KIRTRLFENL RMLPHAPGVQ MQPIPEDAMD QESEDEDKVN 400
BtHDAC1 301  GGYTIRNVAR CWTYETAVAL DTEIPNELPY NDYFEYFGPD FKLHISPSNM TNQNTNEYLE KIKQRLFENL RMLPHAPGVQ MQAIPEDAIP EESGDEDEED 400
HsHDAC1 301  GGYTIRNVAR CWTYETAVAL DTEIPNELPY NDYFEYFGPD FKLHISPSNM TNQNTNEYLE KIKQRLFENL RMLPHAPGVQ MQAIPEDAIP EESGDEDEDD 400

RmHDAC1 401  PDERISIRAS EKRVACDEEF SDSEDEGEGR RDNRSHRPKK HRKGPAPGSA GAATPGANAA ATPGGGAAVA PSPTGGDDEA RTSPAVDVKA AVPAGGDGPQ 500
BtHDAC1 401  PDKRISICSS DKRIACEEEF SDSDEEGEGG RKNSSNFKKA KR-------- ---------- ---------- ---VKTEDEK EKDP--EEKK EVTEEEKTKE 467
HsHDAC1 401  PDKRISICSS DKRIACEEEF SDSEEEGEGG RKNSSNFKKA KR-------- ---------- ---------- ---VKTEDEK EKDP--EEKK EVTEEEKTKE 467

RmHDAC1 501  ALKKDVDDKD DKKMDTSSSD KDAKP 525
BtHDAC1 468  EKQEAKGVKE EVKLA----- ----- 482
HsHDAC1 468  EKPEAKGVKE EVKLA----- ----- 482
```

J

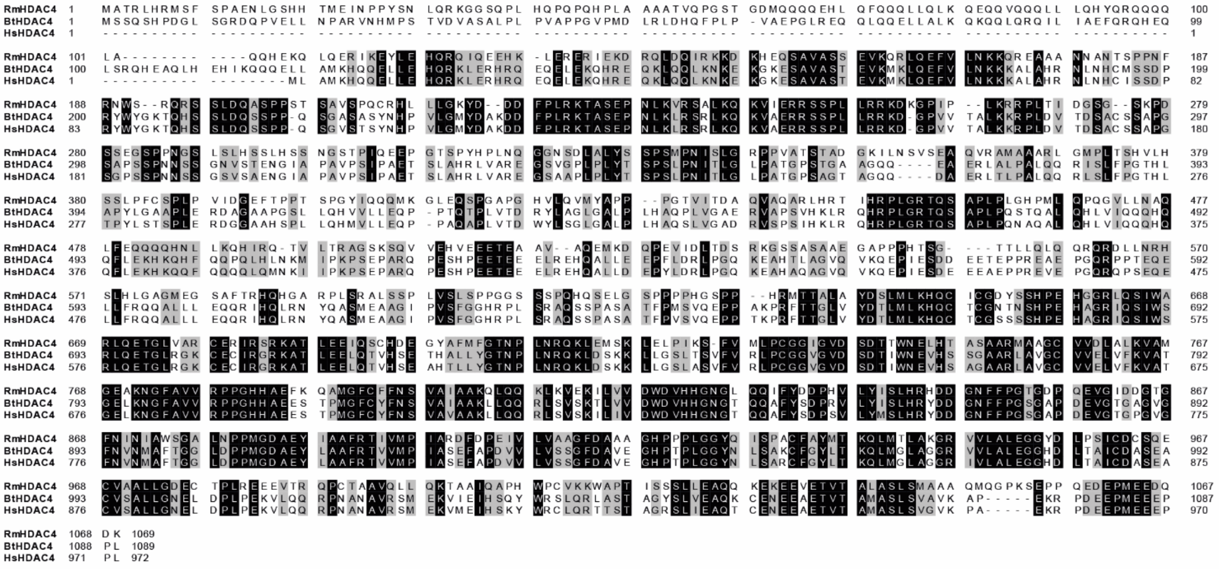

K

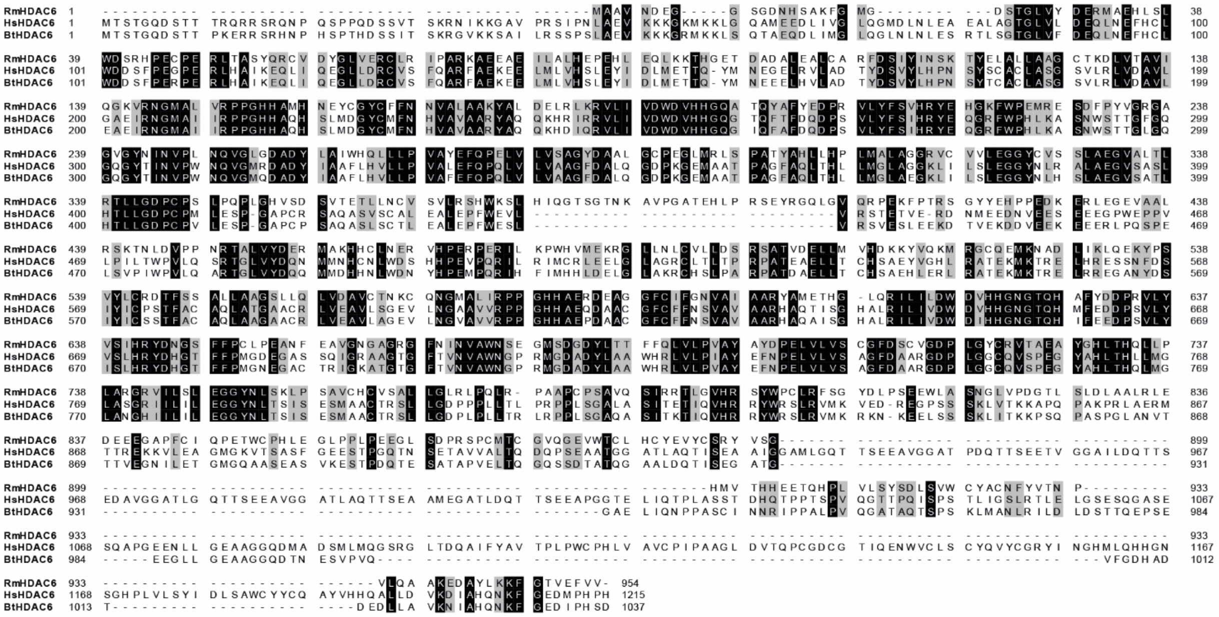

L

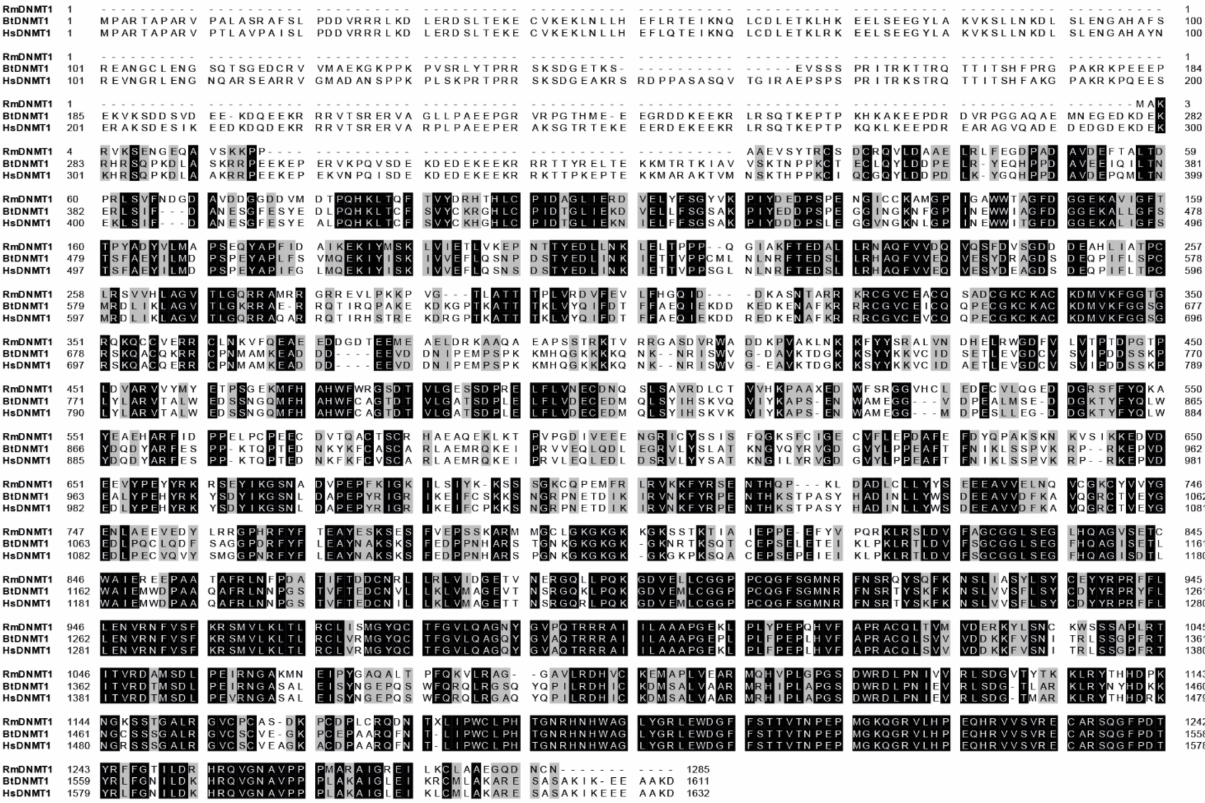
RmDNMT1
BtDNMT1
HsDNMT1

M

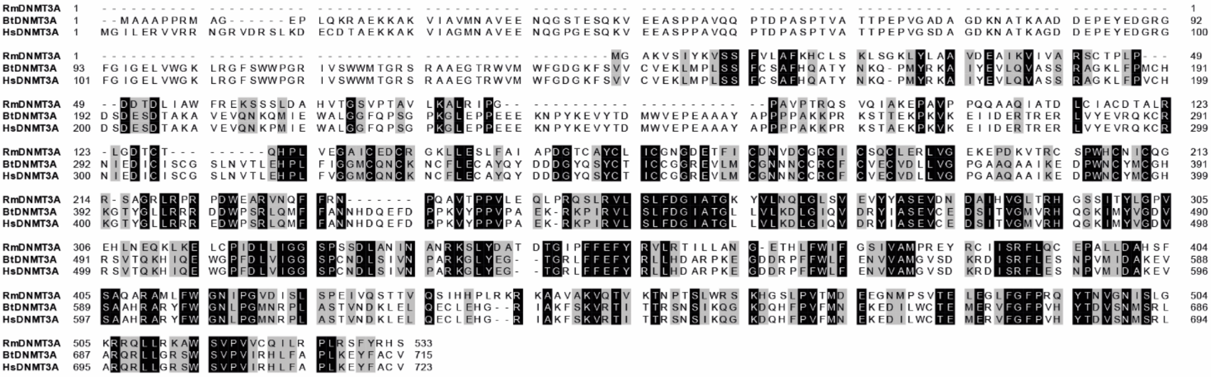
RmDNMT3A
BtDNMT3A
HsDNMT3A

N

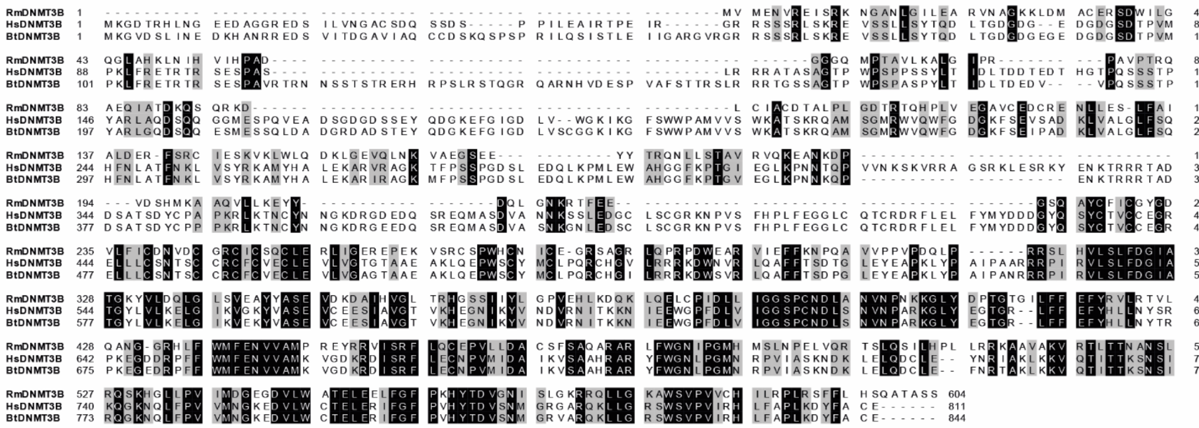
RmDNMT3B
HsDNMT3B
BtDNMT3B

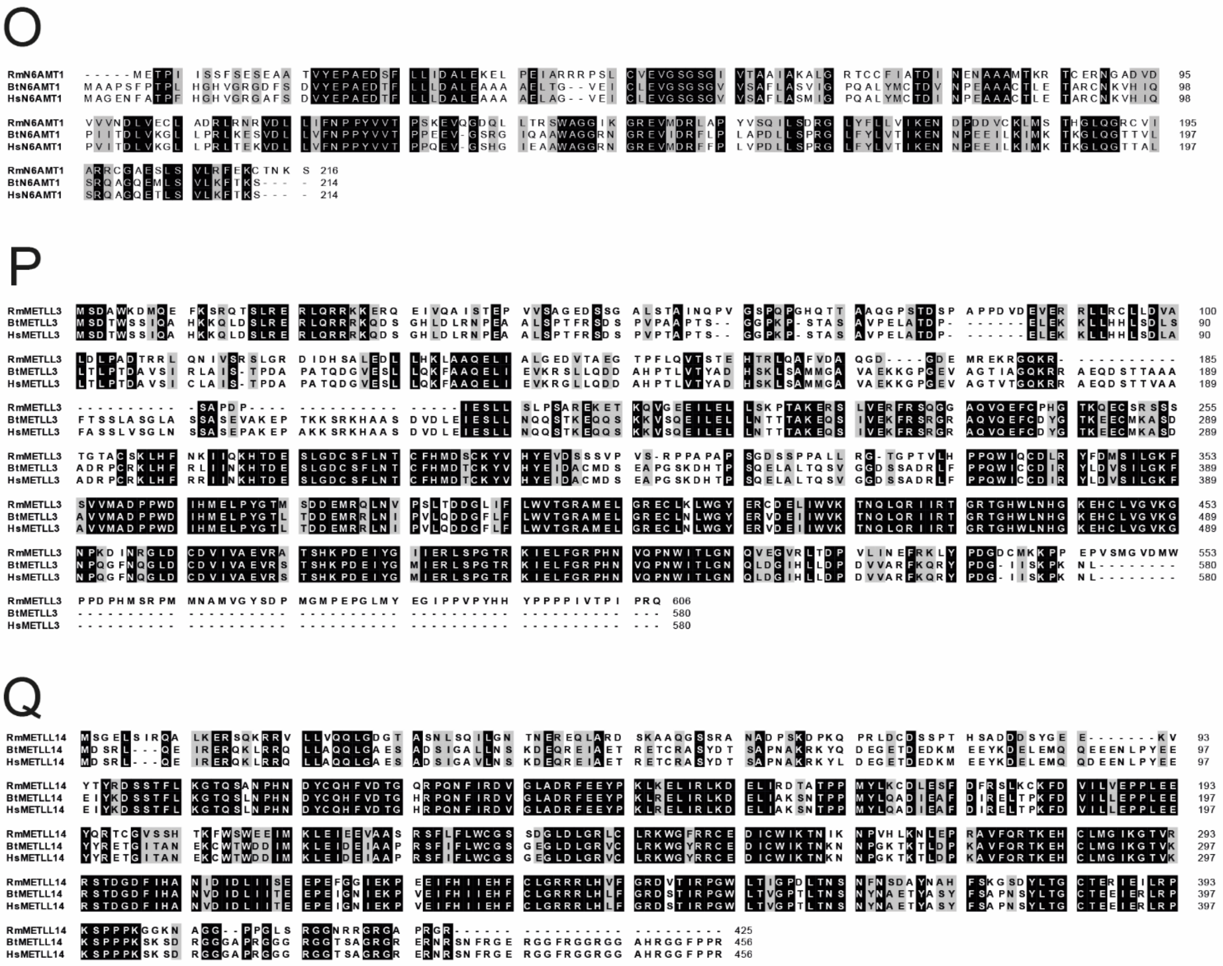
O
RmN6AMT1
BtN6AMT1
HsN6AMT1
P
RmMETLL3
BtMETLL3
HsMETLL3
Q
RmMETLL14
BtMETLL14
HsMETLL14
