# Supplementary material for: Unravelling the role of epigenetic regulators during embryonic development of Rhipicephalus microplus
Source: bioRxiv. 2025 Jul 11:2025.07.11.662657. Preprint. [Version 1] doi: 10.1101/2025.07.11.662657 (PMC12265629; doi:10.1101/2025.07.11.662657)
Supplement: Supplement 2 — Supplemental Figure 2. (A,B). Phylogenetic trees using Ezh and CBP-p300 orthologs across different species, with domain architecture representation. The tree on the left represents evolutionary relationships based on sequence similarity. Bootstrap support values are indicated at each node. On the right, colored bars represent conserved protein domains, as annotated in the legend. The highlighted sequence from Rhipicephalus microplus indicates our species of interest. Domain annotations are based on Pfam. Supplemental Figure 3. SDS-PAGE and Western blot analysis of total protein extracts from Rhipicephalus microplus eggs. Eggs from days 1 (1D) to 21 (21D) were probed for the presence of histones H3 and H4 using monoclonal antibodies. Histone levels are notably absent at days 1 and 3, as indicated by the lack of corresponding bands. Supplemental Figure 4. Detection of m6A methylation in genomic DNA of Rhipicephalus microplus. (A) Agarose gel electrophoresis of genomic DNA isolated from BME26 cells and eggs. The upper band (arrow) corresponds to genomic DNA, while the lower band (arrow) indicates RNA contamination. Treatment with RNase A effectively removed RNA contaminants (lanes 3 and 6). (B) Genomic DNAs from cells or eggs that were treated with RNase A were evaluated by Dot blot analysis showing the presence of m6A methylation in RNA-free genomic DNA. Supplemental Figure 5. Lack of 5-methylcytosine (m5C) modification in total RNA isolated from Rhipicephalus microplus. (A, C) Agarose gel electrophoresis of total RNA isolated from BME26 cells (A) and embryos collected from day 3 to day 21 (C), confirming RNA integrity. (B, D) Dot blot analysis showing no detectable m5C methylation in RNA from BME26 cells (B) or embryos (D). Genomic DNA from the same samples was used as a positive control, demonstrating the presence of m5C DNA methylation (gDNA). Supplemental Figure 6. Hyperacetylation of histones in BME26 cells. (A) BME26 cells were treated with 100 μM TSA for 48 hou [file media-2.pdf]

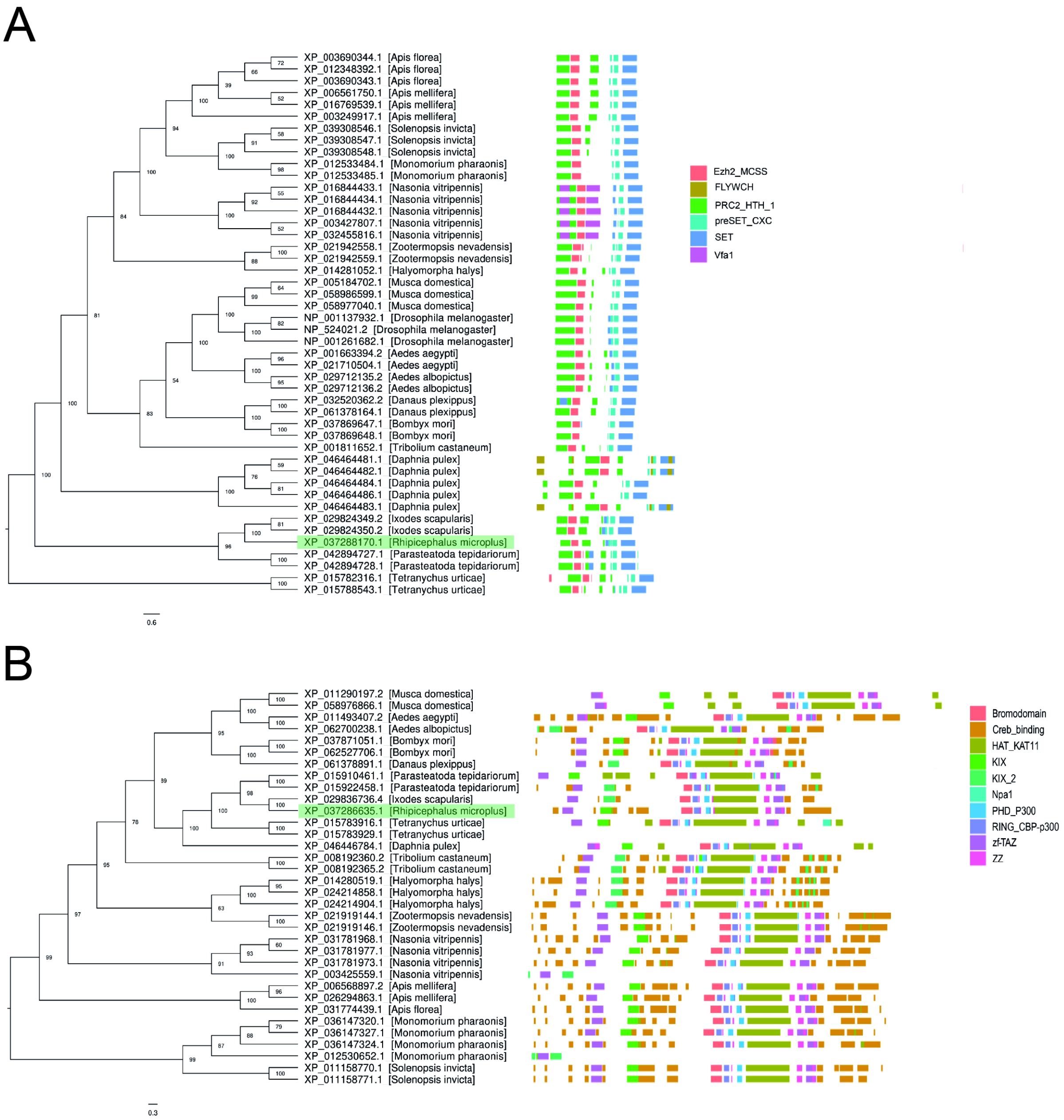

Supplemental Figure 2.

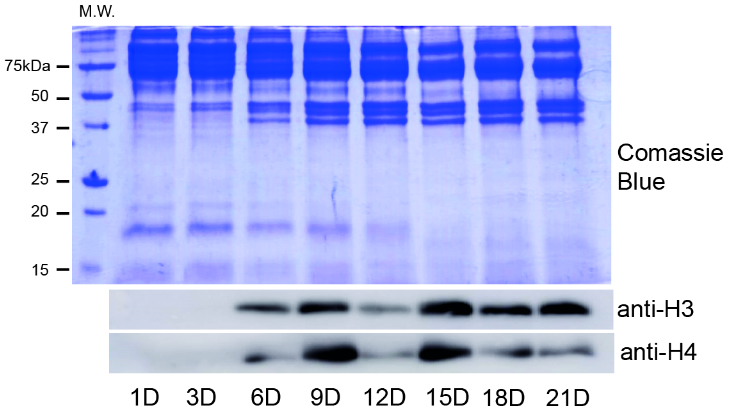

Supplemental Figure 3.

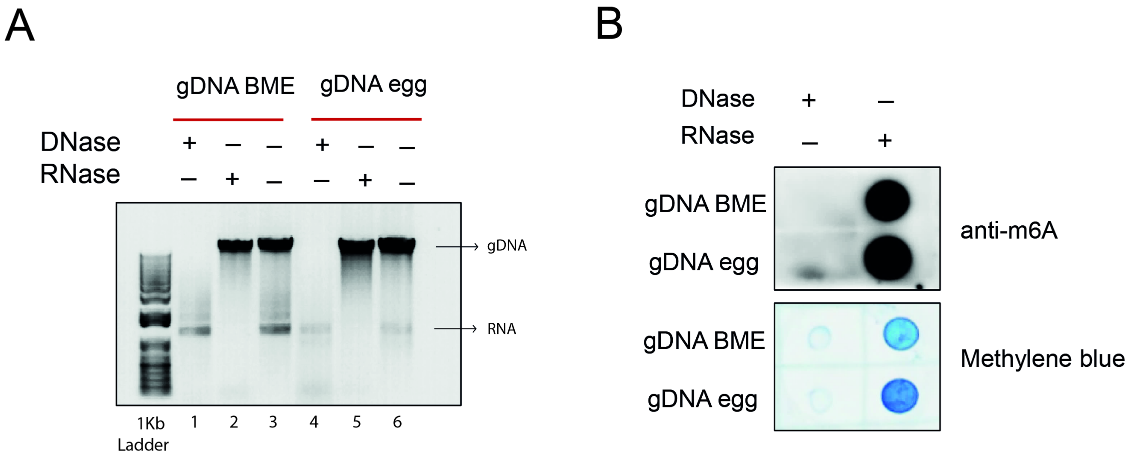

Supplemental Figure 4.

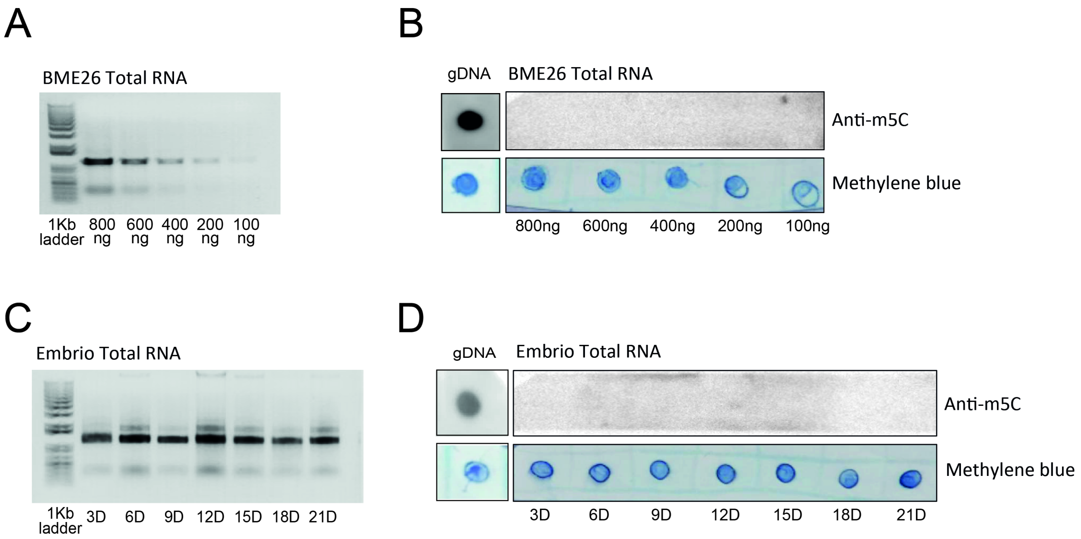

Supplemental Figure 5.

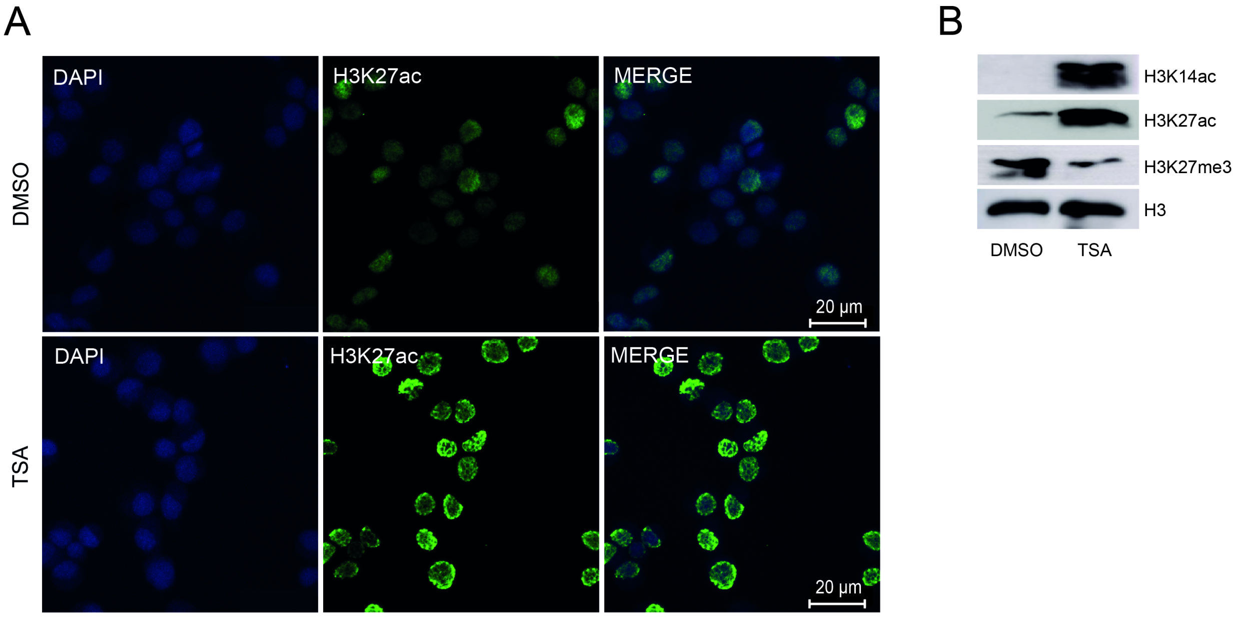

Supplemental Figure 6.

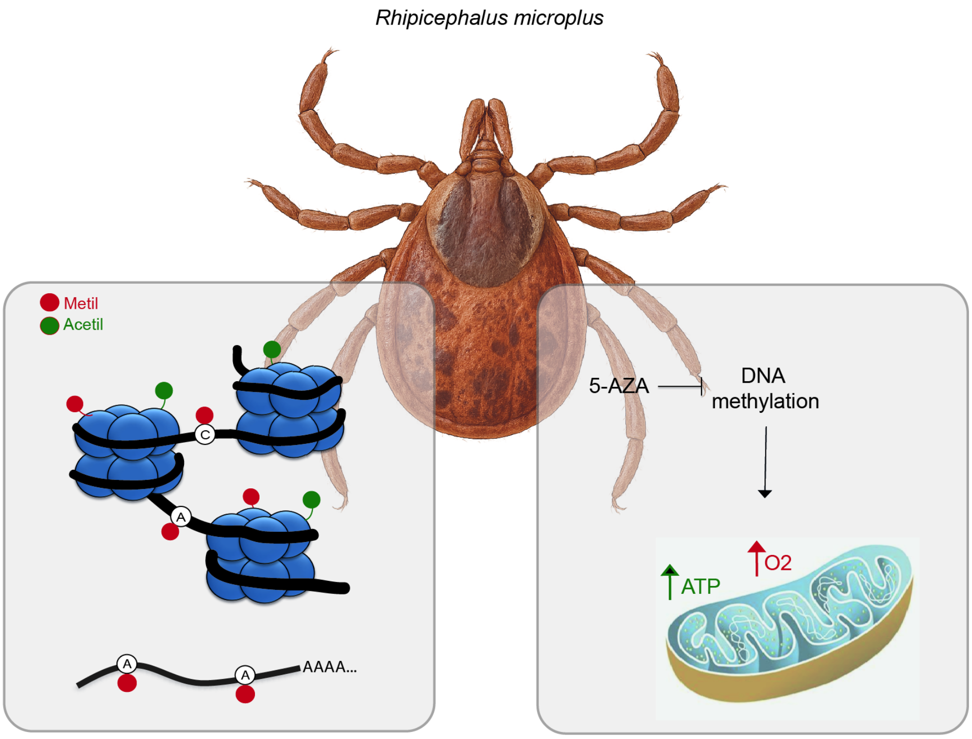

Graphical Abstract
